# Treatment with synthetic β-lactoglobulin peptides can prevent clinical symptoms in a mouse model for cow’s milk allergy

**DOI:** 10.1186/2045-7022-3-S3-P83

**Published:** 2013-07-25

**Authors:** L Meulenbroek, B van Esch, G Hofman, A Nauta, L Willemsen, C Bruijnzeel-Koomen, J Garssen, E van Hoffen, L Knippels

**Affiliations:** 1 Pharmacology, Utrecht Institute for Pharmaceutical Sciences, Utrecht, the Netherlands; 2 Dermatology/Allergology, University Medical Center Utrecht, Utrecht, the Netherlands; 3 Immunology, Danone Research Centre for Specialised Nutrition, Wageningen, the Netherlands

## Background

Previous animal studies showed that partial whey hydrolyates could prevent acute allergic symptoms in a mouse model for cow’s milk allergy. This effect was even more pronounced in combination with a diet containing a specific non-digestible oligosaccharide mixture of sc-Galacto- (scGOS), lc-Fructo- (lcFOS) and Acidic- (pAOS) oligosaccharides (in a 9:1:2 ratio). We hypothesized that these hydrolysates contain peptides that can induce the observed tolerance. Therefore we investigated the potential of synthetic β-lactoglobulin peptides, with or without a diet containing ScGOS/lsFOS/pAOS, to induce tolerance in this mouse model.

## Methods

Female C3H/HeOuJ mice were treated for six consecutive days with three synthetic β-lactoglobulin peptide mixes or PBS. The peptides were selected based on human T cell proliferation data and previous literature. During this treatment period, mice were fed a control or sc GOS/lcFOC/pAOS containing diet. Subsequently, mice were sensitized five times with cholera toxin alone or in combination with whey and received an intradermal ear and oral challenge 6 days later.

## Results

Treating the mice with peptide mix 1 and 3 before sensitization reduced the acute allergic ear response. Peptide mix 2 showed no effect. The effect of peptide mix 1 was stronger in combination with the scGOS/lcFOS/pAOS containing diet. No additional effects were observed for the other mixes. Some mice showed reduced antibody responses but no association with clinical responses was observed. Of the peptides in mixture 1, one peptide showed the strongest effect on the acute allergic skin response. This peptide also tends to decrease whey-specific antibody levels and to increase the percentages of CD11b+CD103+ dendritic cells and CD25+Foxp3+ T cells in the MLN.

## Conclusion

Pre-treatment with specific β-lactoglobulin peptides is able to reduce the acute allergic response in a mouse model for cow’s milk allergy suggesting that specific peptides are capable of inducing tolerance which may involve regulatory dendritic and T cells. The tolerizing capacity could be enhanced by the addition of a scGOS/lcFOS/pAOS containing diet. Further research is necessary to determine whether these peptides are present in the tolerance inducing hydrolysates and the mechanism of action of these peptides should be further investigated.

## Disclosure of interest

L Meulenbroek: None declared, B van Esch: Employee of Danone Research Centre for Specialised Nutrition, G Hofman: None declared, A Nauta: Employee of Danone Research Centre for Specialised Nutrition, L Willemsen: None declared, C Bruijnzeel-Koomen: None declared, J Garssen: Employee of Danone Research Centre for Specialised Nutrition, E van Hoffen: None declared, L Knippels: Employee of Danone Research Centre for Specialised Nutrition.

